# Gastric transposition as a valid surgical option for esophageal replacement in pediatric patients: experience from three Italian medical centers

**DOI:** 10.1093/gastro/gow012

**Published:** 2016-05-04

**Authors:** Rossella Angotti, Francesco Molinaro, Carmine Noviello, Giovanni Cobellis, Ascanio Martino, Carmine Del Rossi, Adrian Bianchi, Mario Messina

**Affiliations:** 1 Division of Pediatric Surgery, Department of Medicine, Surgical and Neurological Sciences, University of Siena, Siena, Italy; 2 Pediatric Surgery Unit, Academic Salesi Children Hospital, Marche Polytechnic University, Ancona, Italy; 3 Department of Pediatric Surgery, University of Parma, Parma, Italy; 4 Neonatal and Paediatric Reconstructive Surgery, Royal Manchester Children’s Hospital, Manchester, UK

**Keywords:** gastric transposition, children, esophageal atresia, caustic ingestion

## Abstract

**Background:** Esophageal replacement in children is an option that is confined to very few situations including long-gap esophageal atresia and esophageal strictures unresponsive to other therapies (peptic or caustic ingestion). The purpose of our work was to describe the experience of gastric transposition in three Italian centers.

**Methods:** This is a retrospective study. The data were extrapolated from a prospective database. We included all patients who had undergone gastric transposition in the last 15 years.

**Results:** In the 15-year period, eight infants and children (3 males and 5 females) underwent gastric transposition for esophageal replacement. Six patients had long-gap esophageal atresia, and two had caustic esophageal stenosis. There were no deaths in the series. Three patients had an early postoperative complication: two had a self-limited salivary fistula at three weeks, and one (a patient with jejunostomy) had a jejunal perforation treated surgically. One late complication, anastomotic stricture, was recorded that required two endoscopic dilatations. The median follow-up was 60 months (range: 18–144 months). At final clinical follow-up, six patients had no eating problems, and two patients had some difficulties with eating (jejunostomy in situ), but they underwent logopedic therapy with improved outcomes. All patients had an increase in body weight and height postoperatively.

**Conclusion:** Our small study reports the clinical experience of three Italian centers in which gastric transposition was performed with excellent results, both in terms of surgical technique (simplicity, reproducibility, complication rate) and clinical follow-up (good oral feeding of young patients, normal social life and regular growth curves).

## Introduction

Esophageal replacement in children is an option confined to few situations: long-gap esophageal atresia (EA) and esophageal strictures unresponsive to other therapies (peptic or caustic ingestion) [1,2]. The turnaround in the past decades has depended on the increased trend of safeguarding the native esophagus [1]. The improvement of surgical techniques for long-gap EA, the evolution of anti-reflux surgery and the major attention to the management of caustic ingestion have played a key role. The purpose of our work was to describe the clinical experience of gastric transposition in three Italian centers.

## Methods

This is a retrospective study conducted at the pediatric surgery centers of Siena, Ancona and Parma. The data have been extrapolated from a prospective database. We included all patients who had undergone gastric transposition in the last 15 years. Sex, age, indication for replacement, age of surgery, associated disorders, preoperative and postoperative management, surgical technique, intraoperative and postoperative complications (early and late) and follow-up were recorded and considered.

## Results

In the 15-year period (from 1999 to 2014), eight children (three boys and five girls) underwent gastric transposition for esophageal replacement. Six patients had long-gap esophageal atresia (EA), and two had caustic esophageal stenosis (Table 1

**Table 1.** Demographic data of the patients and their backgrounds

Sex	Diagnosis	Background	Age at replacement
Female	EA-I	Gastrostomy – first day of life	4 years
		Peritonitis for gastrostomy dislodgement – fifth day of life	
		Esophageal anastomosis – 4 months	
		Esophageal stenosis – 8 months	
		Bowel occlusion, intestinal resection and colonic anastomosis – 3 years	
Female	EA-I	Replogle tube and gastrostomy – first day of life	4 months
Male	EA-III	Closure of TEF, Replogle tube and gastrostomy – first day of life	4 months
Male	EA-III	Closure of TEF, end-to-end anastomosis, gastrostomy	6 years
		Recurrent TEF and esophageal stenosis unresponsive to endoscopic treatment	
		Dor fundoplication – 2 years	
Female	EA-III	Esophageal anastomosis and closure of TEF – first day of life	2 years
		Anastomotic leak, cervical esophagostomy and gastrostomy – first week of life	
		Coloplasty – 1 year	
		Complicated to graft’s necrosis, jejunostomy and cervical esophagostomy	
Male	EA-III	Closure of TEF, cervical esophagostomy and gastrostomy – first day of life	15 months
Female	Caustic esophageal stenosis	Caustic ingestion – 20 months	3 years
		Gastrostomy – 24 months	
		Esophageal dilatations complicated with rupture of esophagus	
Female	Caustic esophageal stenosis	Caustic ingestion – 10 years	11 years
		Esophageal dilatations for 6 months	

**Figure 1.**
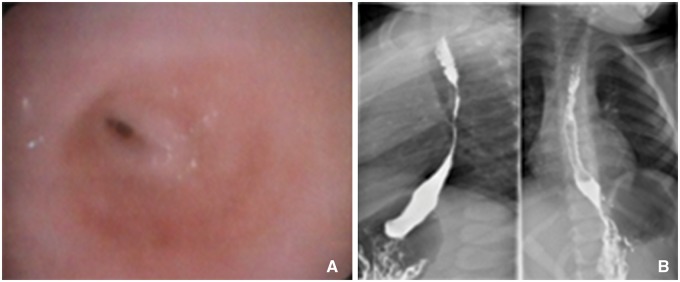
A 3-year-old female patient with an accidental caustic ingestion: (**A**) shows an endoscopic picture of esophageal stenosis, and (**B)** shows an x-ray image of esophageal stenosis.

EA: esophageal atresia; TEF: tracheo-esophageal fistula

). Endoscopic and radiologic images of a patient with esophageal stenosis are shown in Figure 1

.

Of the six children with EA, four had an EA III-type, and two had EA I-type according to the classification of Ladd [3]. Two had urogenital malformations: imperforate anus (male) and cloaca (female). One female was suffering from a complex syndrome characterized by mental retardation, dysmorphia and kidney abnormalities. One male had a cardiovascular anomaly of epiaortic vessels (left common carotid, reduced lumen of proximal aorta). All children underwent different preoperative protocols according to the center in which they were treated. Retrospective analysis of data showed an average preoperative weight of 10 kg (range: 2.4–24 kg) and an average preoperative height of 92 cm (range: 50–140 cm). All patients were below the third percentile for weight and height related to their age.

**Figure 2.**
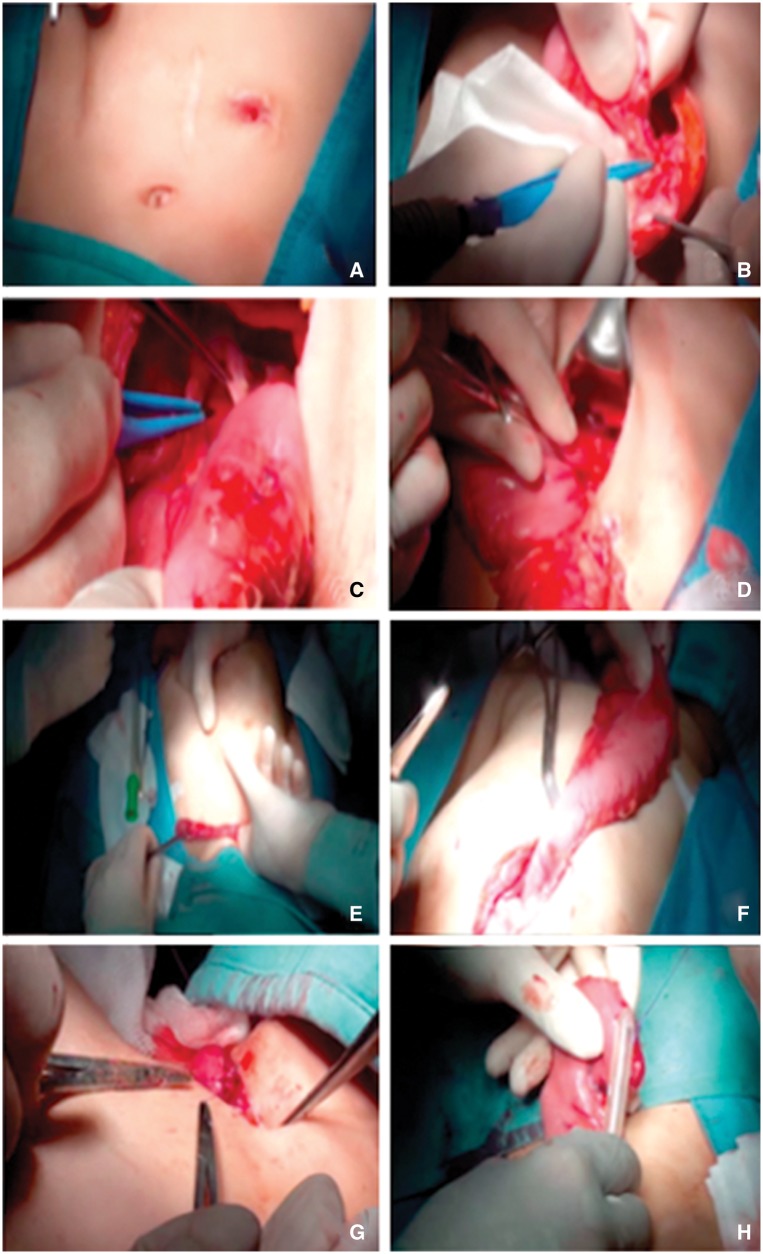
Main steps of gastric transposition for esophageal atresia: (**A)** midline upper abdominal incision and elliptical incision around the cervical esophagostomy; (**B)** exposition of the stomach, closure of the gastrostomy; mobilization of greater and lesser curvatures of the stomach; (**C)** division of esophagogastric junction and repair of the defect in the stomach; (**D)** pyloroplasty; (**E)** dissection for the mediastinal tunnel; (**F)** pulling up of the stomach through the posterior mediastinal tunnel until the fundus appears at the cervical incision; (**G)** mobilization of the esophagus; (**H)** anastomosis between the end of the cervical esophagus and the top of the fundus of the stomach.

Two patients with caustic esophageal stenosis underwent esophagogram and endoscopy to characterize their esophageal morphology and the length and entity of stenosis and to ensure the indemnity of the stomach. The surgical technique of gastric replacement, which has been performed in all centers, was the classical technique via posterior mediastinum. Figure 2

summarizes the main steps of gastric transposition that have been described by Spitz [1,4]. Both pyloromyotomy and abdominal incision were performed in all patients. Thoracotomy was required in five patients and cervical incision in three patients.

Six patients (5 EA and 1 caustic stenosis) had preoperative gastrostomy that had been closed during surgery. One EA patient had a preoperative jejunostomy that was left in place after the gastric replacement. This patient had undergone colonic replacement with necrosis of graft, esophagostomy and jejunostomy. A jejunostomy was placed postoperatively in 4 EA patients who had never eaten orally at normal times and volumes.

There were no deaths in the series. Three patients (37.5%) had early postoperative complications: two had self-limited salivary fistula at three weeks, and one had jejunal perforation (in patients with jejunostomy) and was treated surgically. One late complication was recorded (12.5%): an anastomotic stricture that required two endoscopic dilatations. There was no standardized follow-up. The median follow-up was 60 months (range: 18–144 months). Two patients maintained follow-up to 12 months, two patients to 24 months and four patients to 5 years. At the final clinical follow-up, six patients had no eating difficulties with both liquid and solid foods; two patients still have jejunostomy because of difficulties with eating. However, both of them are undergoing logopedic therapy with improved results. Indeed, we are planning to remove the jejunostomy at the next follow-up. All patients had gain of body weight and height postoperatively.

## Discussion

Based on a retrospective analysis of our experience, we are aware that this study has a number of limits. The group was small (only eight patients), the follow-up (median 60 months) was short, the series were not uniform, and the patients underwent different preoperative and postoperative management. However, the main aim of this study was to report our limited experience in the context of pediatric surgery in an Italian landscape in which the colon is still the first choice for an esophageal substitute. We want to show the feasibility and the favorability in terms of several aspects of the stomach as an esophageal substitute and share our experience with the medical community.

Although we agree with the principle that the child’s own esophagus is the best option and that the esophagus should be preserved in a majority of cases, we are aware that in some situations repeated attempts to preserve the native esophagus may be detrimental to the child, causing multiple residual discomforts.

Esophageal replacement, regardless of types of substitute, remains a major surgery for restoring a ‘pseudonormal’ continuity of the digestive tract and has a high complication rate. To date, there is no suitable organ to replace esophagus with all of its features and functions. Each possible esophageal substitute has pros and cons, and each surgical technique has its benefits, difficulties, complications and success rate (which often vary depending on the experience of the surgeon).

It is known that an ideal organ to replace the esophagus must meet some specific characteristics, especially in children. Its function should not only be to re-establish the continuity of the alimentary canal from the mouth to the stomach but also to ensure its long-term viability, given the long life expectancy of the pediatric patient. It should not affect cardiac or respiratory function during a child’s growth. Finally, it should allow oral feedings and a normal social life. In our series, the stomach was the second choice in three of eight patients. In two patients, peritonitis made the colon unusable; the third patient underwent coloplasty, but the graft became necrotic.

We did not focus our attention on surgical technique because Spitz and other authors have already described them in detail [1–6]. However, we summarized some important aspects after a critical retrospective analysis.

### When is esophageal replacement appropriate in a pediatric patient?

The international literature is exhaustive about this topic. There is no international consensus, but all pediatric surgeons are aware that the indications for esophageal replacement are long-gap esophageal atresia and unresponsive esophageal stenosis [7–9].

### What is the best route for a ‘new’ esophagus?

Our preferred approach is to use the posterior mediastinum as the best option. In fact, it is the shortest and most direct route for reconnecting the cervical region with the abdominal region; it is also the route with natural position of the esophagus, and it reduces the risk of pulmonary and heart compression. None of our patients reported breathing or cardiac problems. It is known that this maneuver is accomplished with many difficulties in children who have had previous surgery or sepsis in the mediastinum [6].

### Is cervical esophagostomy problematic during replacement?

Right cervical esophagostomy should be the preferred route in all cases. Two of our patients had a left cervical esophagostomy, which caused many problems during replacement surgery. The presence of the aortic arch, indeed, is often a limiting factor. Right cervicostomy is safer and faster. It bypasses the heart and great vessels, making any operation for restoring esophageal continuity much safer.

### Is jejunostomy a good option for the patient? Is it a limit for replacement?

One of our patients had a preoperative jejunostomy, which made the gastric transposition difficult because the excluded stomach was small and non-functional. Jejunostomy is indeed important for children with EA who have never eaten, and it can be a useful tool for managing the nutritional status of a patient even after gastric transposition. However, it is not optimal in view of possible gastric transposition because the jejunal probe bypasses the stomach, making it atrophic and small and causing many intraoperative problems in the cervical region.

### What is good time to perform a replacement?

This is a problematic aspect. There are many variations between patients who need esophageal replacement for esophageal atresia or caustic ingestions. In the esophageal atresia group, it has been reported that early gastric transposition (in the first six months of life) is meaningless because the child does not stay upright so swallowing with a ‘neo-esophagus’ without peristalsis is rather difficult [1–6]. However if neurophysiology of feeding and swallowing are considered and the emphasis is placed on the importance of re-establishing continuity of the digestive tract in the shortest possible time to allow neuronal adaptation to swallowing, it is acceptable as an early gastric transposition. There are many studies of neurophysiology reporting that the first few days/months of life are critical because the totipotent nerve cells acquire specific capacities such as swallowing, defecation, urination, etc [10,11]. The exact timing of this set-up in humans to date is not known, but many studies on the neurophysiology of the brain of animals (such as cats) show that the totipotent cells die without possibility of regeneration if they do not receive adequate stimulus within the first months of life.

Therefore, if the the swallowing reflex a baby with esophageal atresia is not stimulated from birth to, his or her pattern of nerve cells intended to acquire the ability to swallow regress irreversibly, causing multiple problems after surgery to restore esophageal continuity. We are aware that the patients undergoing early gastric transposition may need some maneuvers to facilitate their swallowing, and we are certain that all of these maneuvers have less impact on the child’s life in regard to long-term follow up. In our series, only two children underwent gastric transposition at four months of life, and both had a postoperative jejunostomy, but they have never had problems with eating either solid food or liquid. One of the children had jejunostomy for a long time due to poor compliance of the parents.

The other four patients who underwent replacement after the first year of life had no difficulties in terms of eating. However, it is important to acknowledge that two of them were followed by a logopedist since birth and that they used esophagostomy to learn to eat; The other two underwent esophageal anastomosis in the first month of life and began to eat normally.

As for caustic esophageal stenosis, there is no fixed term to operate on these patients. It is known that esophageal replacement should be the last choice only after failure of ‘conservative’ treatment (medical or endoscopic). As we reported above, we agree with the principle that the child’s own esophagus should be preserved in a majority of cases, but we are aware that, in some situations, repeated attempts to preserve the native esophagus may be detrimental to the child, causing multiple residual discomforts. So, in our opinion, the age of esophageal replacement is when the patient with caustic stenosis has unresponsive esophageal stenosis. In our series, all patients underwent replacement after 1 year of conservative treatment, and they had no problems in term of postoperative feeding because none of them had eating problems.

## Conclusion

Gastric transposition, as stated before, is not particularly popular in children, but its supporters (mainly English) have been increasing in recent years [9,12,13]. Our small study reports the experience of three Italian centers where gastric transposition was performed with excellent results in terms of surgical technique (simplicity, reproducibility, complication rates) and clinical follow-up (good oral feeding of young patients, normal socializing, regular growth curve (height-weight)). The future objective of this study, however, is to perform a national survey to understand the general orientation of Italian pediatric surgeons relative to gastric replacement and to design a diagnostic/therapeutic algorithm for gastric replacement in the Italian pediatric patient.

*Conflict of interest statement:* none declared.

## References

[1] Spitz L Esophageal Replacement. In: Mattei Peter (ed). Fundamentals in Pediatric Surgery. New York: Springer, 2011, 247–52.

[2] Cerchia E Molinaro F Pavone M Esophageal atresia with distal tracheoesophageal fistula: surgery treatment and a long term follow up. J Siena Acad Sci 2012;4:30–4.

[3] Ladd WE The surgical treatment of esophageal atresia and tracheoesophageal fistulas. N Engl J Med 1944;230:625–37.

[4] Spitz L Kiely E Pierro A Gastric transposition in children: a 21-year experience. J Pediatr Surg 2004;39:276–81. 15017537 10.1016/j.jpedsurg.2003.11.032

[5] Cowles RA Coran AG Gastric transposition in infants and children. Pediatr Surg Int 2010;26:1129–34. 20878410 10.1007/s00383-010-2736-9

[6] Arul GS Parikh D Oesophageal replacement in children. Ann R Coll Surg Engl 2008;90:7–12. 10.1308/003588408X242222 PMC2216706 18201490

[7] Mas E Breton A Lachaux A Management of caustic esophagitis in children. Arch Pediatr 2012;19:1362–8. 23141564 10.1016/j.arcped.2012.09.013

[8] Spitz L Esophageal stenosis, stricture and replacement. In: O’Neill J Grosfeld J Fonkalsrud E (eds). Principles of Pediatric Surgery. Philadelphia: Mosby, 2003,395–404.

[9] Loukogeorgakis SP Pierro A replacement surgery for esophageal atresia. Eur J Pediatr Surg 2013;23:182–90. 23720209 10.1055/s-0033-1347915

[10] Bale TL Epigenetic and transgenerational reprogramming of brain development. Nat Rev Neurosci 2015;16:332–44. 25921815 10.1038/nrn3818 PMC7064155

[11] Williams TC Drake AJ What a general paediatrician needs to know about early life programming. Arch Dis Child 2015;100:1058–63. 25990501 10.1136/archdischild-2014-307958

[12] Gupta L Bhatnagar V Gupta AK Long-term follow-up of patients with esophageal replacement by reversed gastric tube. Eur J Pediatr Surg 2011;21:88–93. 21058245 10.1055/s-0030-1267240

[13] Showalter CD Kerrey B Spellman-Kennebeck S Gastrostomy tube replacement in a pediatric ED: frequency of complications and impact of confirmatory imaging. Am J Emerg Med 2012;30:1501–6. 22306396 10.1016/j.ajem.2011.12.014

